# Magnetic resonance imaging at 9.4 T: the Maastricht journey

**DOI:** 10.1007/s10334-023-01080-4

**Published:** 2023-04-20

**Authors:** Dimo Ivanov, Federico De Martino, Elia Formisano, Francisco J. Fritz, Rainer Goebel, Laurentius Huber, Sriranga Kashyap, Valentin G. Kemper, Denizhan Kurban, Alard Roebroeck, Shubharthi Sengupta, Bettina Sorger, Desmond H. Y. Tse, Kâmil Uludağ, Christopher J. Wiggins, Benedikt A. Poser

**Affiliations:** 1 Faculty of Psychology and Neuroscience, Maastricht University, Universiteitssingel 40, 6229 ER Maastricht, The Netherlands; 2 Institute of Systems Neuroscience, Center for Experimental Medicine, University Medical Center Hamburg-Eppendorf (UKE), Hamburg, Germany; 3 Krembil Brain Institute, University Health Network, Toronto, ON Canada; 4 Philips Healthcare, Best, North Brabant The Netherlands; 5 Scannexus BV, Oxfordlaan 55, 6229 EV Maastricht, The Netherlands; 6 Krembil Brain Institute, Koerner Scientist in MR Imaging, University Health Network Toronto, Toronto, ON Canada; 7 Department of Medical Biophysics, University of Toronto, Toronto, ON Canada; 8 Center for Neuroscience Imaging Research, Institute for Basic Science and Department of Biomedical Engineering, Sungkyunkwan University, Suwon, Republic of Korea; 9 Imaging Core Facility (INM-ICF), Institut für Neurowissenschaften und Medizin, Forschungszentrum Jülich GmbH, 52425 Jülich, Germany

**Keywords:** Ultra-high field, 9.4 T, fMRI, pTx

## Abstract

The 9.4 T scanner in Maastricht is a whole-body magnet with head gradients and parallel RF transmit capability. At the time of the design, it was conceptualized to be one of the best fMRI scanners in the world, but it has also been used for anatomical and diffusion imaging. 9.4 T offers increases in sensitivity and contrast, but the technical ultra-high field (UHF) challenges, such as field inhomogeneities and constraints set by RF power deposition, are exacerbated compared to 7 T. This article reviews some of the 9.4 T work done in Maastricht. Functional imaging experiments included blood oxygenation level-dependent (BOLD) and blood-volume weighted (VASO) fMRI using different readouts. BOLD benefits from shorter T_2_* at 9.4 T while VASO from longer T_1_. We show examples of both ex vivo and in vivo anatomical imaging. For many applications, pTx and optimized coils are essential to harness the full potential of 9.4 T. Our experience shows that, while considerable effort was required compared to our 7 T scanner, we could obtain high-quality anatomical and functional data, which illustrates the potential of MR acquisitions at even higher field strengths. The practical challenges of working with a relatively unique system are also discussed.

## Introduction

The MRI signal is determined by the properties of the MRI sequences and the RF coils utilized, such as timing and shape of the RF pulses and MR gradients, the B_1_^+^ and B_1_^−^ sensitivity profiles, as well as by the local tissue biophysical characteristics. Among those, the equilibrium magnetization, T_1_ and T_2_^(^*^)^ relaxation times are all magnetic field strength dependent. Hence, image contrast and Signal-to-Noise Ratio (SNR) are also MRI sequence- and field strength-dependent. Whilst equilibrium magnetization promises higher SNR with field strength [1, 2], T_1_ and T_2_^(^*^)^ changes can lead to increases in anatomical and functional sensitivity, depending on the acquisition parameters [3]. The SNR of brain images at 9.4 T has been determined to be 1.76 times the 7 T levels [1], and the sensitivity to key MRI attributes of tissue (T_1_, T_2_* and magnetic susceptibility) properties also increases. This is particularly the case for the sensitivity to myelin and iron, which are important markers of neurodegeneration and neurodevelopment. In addition, an at least linear increase in blood oxygenation level-dependent (BOLD), which forms the basis of most fMRI of the brain, effect size is theoretically expected [4] and has been experimentally observed [5–8]. Both effects, higher image SNR and larger BOLD effect, independently improve the contrast-to-noise ratio (CNR), leading to more than a doubling of the BOLD CNR ratio from 7 to 9.4 T. This has allowed researchers on our sister system in Tubingen to push to higher spatial resolution at 9.4 T beyond what has been feasible at 7 T [9].

With the neuroscientific focus in Maastricht, we rely heavily on echo-planar imaging (EPI) sequences, and a head-gradient set was thus chosen for our Siemens system with passively shielded Magnex whole-body magnet. The passively shielded 9.4 T system is part of the Brains Unlimited project including actively shielded whole-body 7 T and 3 T systems by the same vendor. The project was initiated by Rainer Goebel and the funding was provided by the university and the regional government. The system was delivered in May 2013 and first in vivo images were acquired in September 2013. The head-gradient set allows for the rapid gradient switching needed for EPI readouts as well as diffusion imaging. This keeps echo times relatively short for harnessing the SNR gains, while meeting the desire for higher spatial resolutions and countering the shortening of T_2_* and T_2_ at 9.4 T.

Radio frequency (RF) inhomogeneity is one of the most challenging aspects of UHF MRI. At 3 T or above, the RF wavelength in tissues becomes comparable to, or shorter than, the dimension of the body part being imaged [10–17]. This leads to interferences in the transmitted RF (B_1_^+^) field and results in strong intensity and contrast variations in the final image [16]. The severity of the RF inhomogeneity increases from 7 T to 9.4 T [17] as the RF wavelength decreases. RF inhomogeneity can be addressed in several ways: customized RF coil design [10], dielectric pads [18, 19], tailored RF pulse design [20] as well as using parallel RF transmission (pTx)—B_1_^+^ shimming [17, 21–23] and transmit sensitivity encoding (Transmit-SENSE) [24, 25].

The change in relaxation times and the increased effects of magnetic susceptibility at UHF have both advantages and disadvantages. On the one hand, they enhance various imaging contrasts, but on the other hand the elevated static field (B_0_) inhomogeneity causes stronger image distortions [26], blurring and signal loss [27]. A strong spatial variation of the B_0_ field also has an adverse effect on RF pulse designs that are required to mitigate the RF inhomogeneity at UHF. In a standard MR system, 1st and 2nd orders shim coils are used to reduce B_0_ inhomogeneity. At UHF, higher-order shim coils such as 3rd and 4th orders can be added to further improve both global and local B_0_ homogeneity [28]. At our 9.4 T scanner, an in-house B_0_- and flip-angle-homogenisation workflow (described in Sect. pTx workflow below) was used to address the aforementioned challenges of UHF MR [29]. This was necessary to work around the limitation that the vendor-provided procedures do not fully support pTx mode operation and a better control over the B_0_ and B_1_ adjustments is needed. Another major obstacle compared to the local 7 T scanner initially was the lack of vendor-provided RF-coils that could be immediately employed.

This paper presents a review of various efforts in anatomical and fMRI methods development, post mortem brain sample imaging and cognitive neuroscience applications from multiple researchers and groups at Maastricht University. Some of the work has been previously published, while part is presented for the first time. Nevertheless, the paper does not attempt to cover all the work that has been performed at our scanner but rather give an overview of the different directions researchers have taken to utilize the additional gains at 9.4 T and the methodological advances necessary to make the endeavors a success. The challenges come from exacerbation of SAR and B_1_^+^ inhomogeneities, which require the use of pTx in order to harness the SNR potential available: without optimized RF coils and dedicated transmit strategies, the benefit of 9.4 T over 7 T might not be realized. Therefore, the team in Maastricht explored pTx strategies to support its diverse interests in imaging the brain [29–31]. The paper will conclude with a summary and a discussion about our experiences with the 9.4 T scanner.

All work shown in this paper was acquired on the Maastricht whole-body 9.4 T human MR scanner (Siemens Healthineers, Erlangen, Germany) using a head gradient set (AC84-mk2, maximum amplitude 80 mT/m, maximum slew rate 333 T/m/s, inner diameter 36 cm) in combination with a 16-channel parallel transmission system (1 kW per channel) and several custom RF coils. The various projects presented below utilized different RF coils, and other hardware, software, acquisition, processing and analysis specifics that are described in the respective sections.

## pTx workflow

This section describes the pTx workflow that has been developed in Maastricht and has been also employed in several of the other projects described later. The experiments utilized a dual-row 16-channel transmit/31-channel receive array coil [32]. Online local SAR monitoring was achieved by a vendor-provided system installed on the transmit chain [33]. The SAR matrices used for the online local SAR monitoring were derived from an EM simulation [22] on an adult male model (Hugo) with a safety margin of a factor of two and limits set according to [34] and compressed using the virtual observation points (VOPs) method [35]. The same VOPs compressed SAR matrices were used in SAR prediction in the RF pulse calculations and simulations. The B_0_- and flip-angle homogenisation workflow consists of calibration scans, B_0_ shim and RF calculations, and optional B_0_ and flip angle maps validations. All the calculations are carried out in MATLAB (MathWorks, Natick, MA, US).

### ***B***_***0***_*** and B***_***1***_^+^***mapping***

B_0_ field maps were obtained from a dual-echo 3D GE sequence (TR = 30 ms, TE_1_ = 1.00 ms, TE_2_ = 3.21 ms, nominal flip-angle = 8 degrees, nominal voxel size = 4 mm isotropic, matrix size 50 × 50 × 44, bandwidth 1560 Hz/pixel, total scan duration 1:49 min). Optimal B_0_ shim currents for the 1st, 2nd and four 3rd (Z3, Z2X, Z2Y, ZX2Y2)-order shim coils were calculated from the B_0_ map [36, 37]. The B_0_ field spatial distribution after applying the new shim currents is essential in the following step of calculating the flip-angle homogenized parallel transmission pulse. This was either generated from the B_0_ shim current optimisation or from a second B_0_ field mapping scan, which also served as a validation of the results of the B_0_ shim optimisation.

Complex B_1_^+^ transmit maps from all the transmit channels were obtained using a transmit phase-encoded [38], T_2_- and T_2_*-compensated version of DREAM [39] (imaging train repetition time = 6.8 ms, TR = 7.5 s, TE_1_ = 2.22 ms, TE_2_ = 4.44 ms, nominal imaging flip-angle = 7 degrees, nominal preparation pulse flip-angle = 55.5 degrees, imaging slice thickness 4 mm, slice separation 10 mm, preparation pulse slice thickness 8 mm, voxel size 4 mm isotropic, matrix size 64 × 56 × 15, bandwidth 690 Hz/pixel, 32 transmit phase encode steps, total scan duration 4:00 min). These individual transmit channel B_1_^+^ maps and the B_0_ map after shimming were used as the inputs to the parallel transmission RF pulse optimisation.

The flip-angle distribution achieved by the optimised parallel transmission RF pulse was mapped with a pre-saturation turbo-flash (PreSat-TFL) sequence [40] (imaging train repetition time 5.9 ms, TR 10 s, TE 2.24 ms, nominal imaging flip-angle = 8 degrees, nominal preparation pulse flip-angle 45 degrees, imaging slice thickness 4 mm, voxel size 4 mm isotropic, matrix size = 64 × 64 × 1, bandwidth 690 Hz/pixel, total scan duration 20 s). This was used to validate the RF pulse optimisation results.

### RF pulse calculation

The parallel transmission RF pulses utilised were designed using the spatial domain method [24] with magnitude least square optimisation [41] under the small tip-angle approximation. The optimisation was done using a conjugate-gradient based algorithm [42], which includes a global SAR regularisation and a local SAR regularisation by means of a VOP-compressed SAR matrix [43]. k_T_-points [44] were used as the k-space trajectory for non-volume-selective pulses and spokes for [45] slice- or slab-selective pulses. Static B_1_^+^ shimming, which can be considered as a special case of single k_T_-point at the k-space centre, was also calculated with the same algorithm.

### ***Application of k***_***T***_***-points to MPRAGE and 3D EPI***

**Fig. 1:**
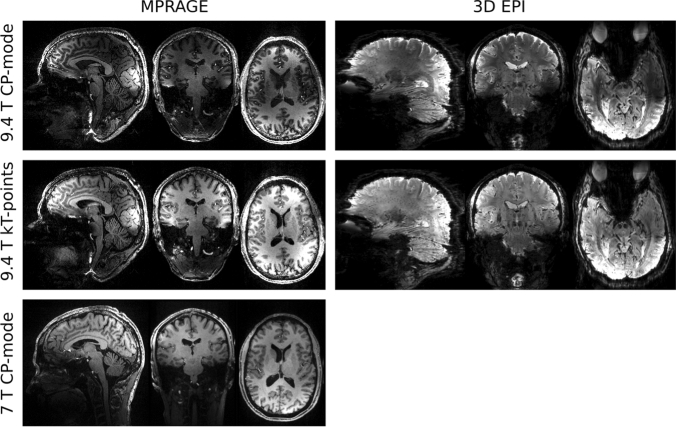
MPRAGE (0.6 mm isotropic resolution) and 3D EPI (0.75 mm isotropic resolution) images, obtained using CP-mode (top row) and k_T_-points (middle row) excitations. MPRAGE (0.6 mm isotropic resolution) from the same participant acquired using the 1Tx/32Rx Nova Medical head coil at 7 T (CP-mode)

Figure 1 shows the 3D imaging examples of MPRAGE and 3D EPI using k_T_-points pulses. The MPRAGE scan was acquired using the following parameters: TR = 3.75 s, TE = 3.64 ms, TI = 1.2 s, matrix size 384 × 384 × 256, voxel size 0.6 mm isotropic, sagittal slices and anterior-to-posterior phase encoding direction, slice partial Fourier 7/8, echo train length = 2.1 s, flip-angle = 5 degrees, GRAPPA factor 3 along the in-plane phase encoding direction with 24 reference lines, bandwidth 180 Hz/pixel. The total scan duration was 8:58 min. The inversion pulse employed was TR-FOCI [46]. The same k_T_-points excitation was also applied in the 3D EPI sequence [47] with parameters as follows: TR = 61 ms, effective volume TR = 12.7 s, TE = 22 ms, matrix size 256 × 256 × 208, voxel size 0.75 mm isotropic, phase partial Fourier 6/8, flip-angle 15 degrees, bandwidth 1396 Hz/pixel, and GRAPPA factor 3 along the in-plane phase encoding direction using 96/48 reference lines/partitions that were acquired in a segmented lines-in-partition order [48]. The total acquisition time was 1:28 min. For a visual comparison, the same images acquired without parallel transmission pulse, i.e. in CP mode, are also shown in Fig. 1, middle row. Finally, an MPRAGE of the same participant with matched acquisition parameters was acquired at our 7 T in combined (CP) mode using the 1 Tx/32 Rx Nova Medical (Wilmington, MA, USA) RF head coil. The direct comparison between the MPRAGE images demonstrates the exacerbation of both the transmit and receive B_1_ inhomogeneity at 9.4 T compared to the 7 T.

### Bipolar spokes

For 2D or slab imaging, flip-angle inhomogeneity can be mitigated by using spokes parallel transmission pulses [45]. In k-space, the spokes pulses’ trajectory follows a series of lines defined by the slice-selective gradient at positions determined by intermittent x–y gradient blips. The slice-selective gradients of these series of pulses can be played out either in the same polarity, or in a more time-efficient manner of alternating polarity, a.k.a. bipolar spokes. It has been shown that in the case of bipolar spokes an undesired time delay $\Delta t$ between the RF pulse and the slice-selective gradient, for example caused by eddy currents, leads to a phase difference $\Delta \phi$ between the odd and even sub-pulses given by the following equation [30]:

1 $$\Delta \phi = 4\pi {\text{BW}}\Delta t\frac{z}{\Delta z},$$

where BW is the bandwidth of the RF pulse, *z* is the slice position from the isocentre and $\Delta z$ is the slice thickness. To overcome this problem in our bipolar spokes slice-selective RF pulses, the phase difference $\Delta \phi$ was compensated in a slice-by-slice manner, or alternatively the starting time of the RF pulse was adjusted to ensure $\Delta t$ was zero [31].

### Spokes pulses for simultaneous multi-slice excitation

**Fig. 2:**
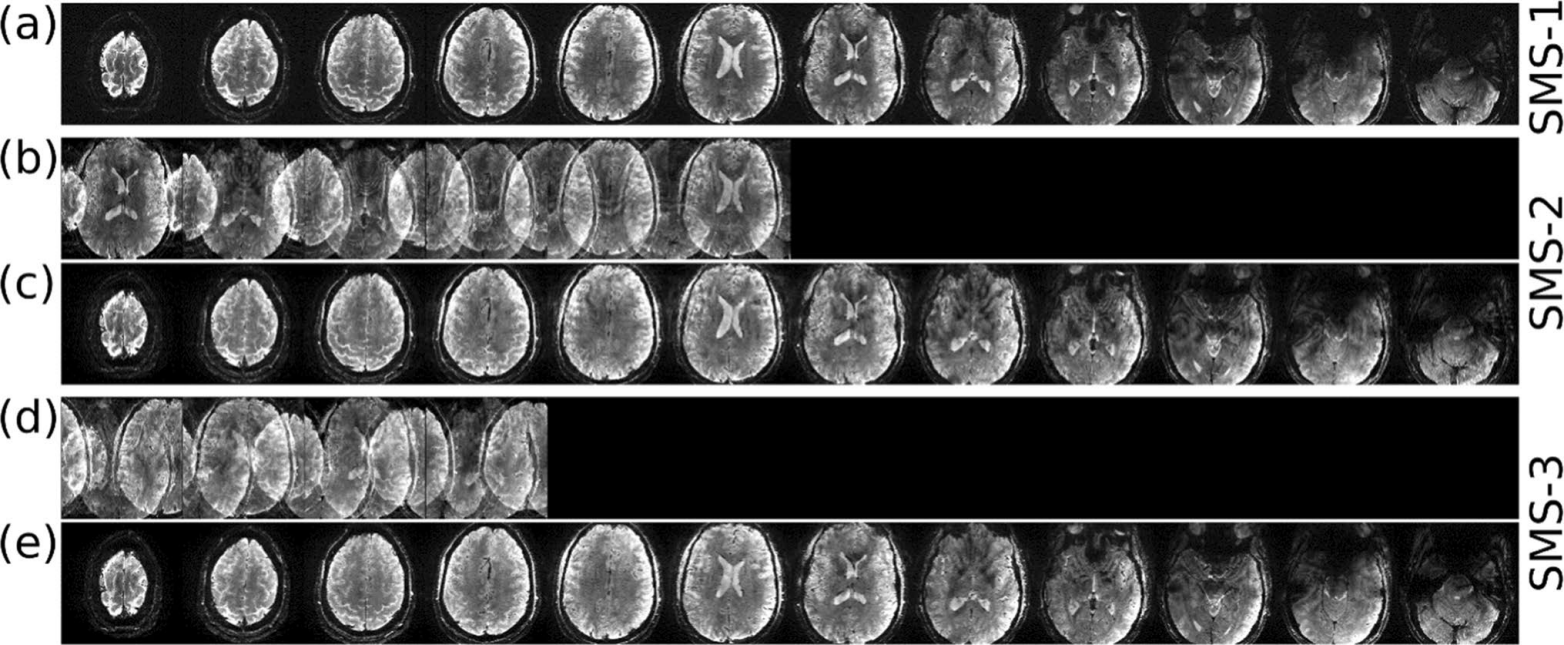
In vivo high-resolution SMS-GE images from [31] acquired with 3-spoke **a** single-band, **b**, **c** SMS-2 and **d**, **e** SMS-3 excitations. Rows **b** and **d** show the SMS images before slice-GRAPPA reconstructionṣ

For multi-slice acquisitions, especially in our case of dual-row 16-channel transmit array coil [32], the best results of within slice flip-angle homogenisation were achieved by slice-wise optimisations of the spokes pulses, i.e. each individual slice had its own optimised complex scaling factor b for each of the spoke sub-pulses. Simultaneous multi-slice (SMS) spokes excitations with slice specific flip-angle homogenisation [31] can be formed by taking the complex scaling factors for each slice, sub-pulse, and transmit channel into account when forming the multi-band pulse by modulating a single-band sinc waveform. The *l*-th time point of the digital multi-band waveform discretised in time for the *j*-th transmit coil and the *k*-th spokes sub-pulse is given as follows:

2 $$f_{{sms_{jkl} }} = \,\sum\limits_{i = 1}^{{N_{sms} }} {{f}_{{\mathrm{sinc}_{l}}}} b_{{i_{jk} }} e^{{i\gamma Gl\Delta tz_{i} }} e^{{i\phi_{i} }} ,$$

where *N*_sms_ is the SMS acceleration factor, ${{f}_{{\mathrm{sinc}_{l}}}}$ is the *l*-th time point of the single band sinc waveform, *b*_*ijk*_ is the complex RF scaling factor of the *j*-th transmit channel and *k*-th subpulse for the *i*-th slice in the SMS group, *G* is the amplitude of the slice-selective gradient, $\Delta t$ is the RF dwell time, *z*_i_ is the slice position of the *i-*th slice in the SMS group, and ${\phi }_{i}$ is an extra slice-dependent phase term that can be used to minimise the peak amplitude of the final multi-band waveform [49, 50]. The slice-dependent phase shift in bipolar spokes mentioned above can be absorbed into *b*_*ijk*_ when forming the multi-band pulse using this equation. Figure 2 shows an example of SMS GE images from [31] with the following imaging parameters: TR = 400 ms, TE = 14 ms, flip angle = 17 degrees, slice thickness = 1 mm, number of slices = 12, in-plane resolution = 0.28 × 0.28 mm^2^, matrix size = 562 × 562 × 12, bandwidth = 70 Hz/pixel, CAIPIRINHA slice shift [51] by gradient blip = FOV/2 for SMS-2 and FOV/3 for SMS-3, no in-plane GRAPPA acceleration. The total acquisition times for the single-band, SMS-2, and SMS-3 protocols were 3:44, 1:52 and 1:15 min, respectively.

## Decoding brain states

One particular fMRI application that can benefit from an increased BOLD sensitivity is decoding brain states based on spatiotemporal BOLD signals evoked by different mental states or activities. Such classification can then be used to create brain–computer interfaces (BCIs) that map intentionally generated mental states to commands. This allows, for example, motor-independent communication or environmental control for patients suffering from the so-called ‘locked-in syndrome’ (LIS). LIS patients have lost their ability to communicate due to severe motor paralysis while maintaining cognitive functions [52]. BCIs that rely on hemodynamic brain signals measured with BOLD fMRI have been shown to provide motor-independent communication via reliably decoded BOLD signals at 3 T [53, 54].

A real-time spelling paradigm was developed by Sorger et al. [55] that allowed encoding of any letter of the alphabet by exploiting the spatiotemporal characteristics of the BOLD signal. In this work, we show the feasibility of such an experiment at 9.4 T using an optimized version of the letter speller. Eight healthy participants (mean age = 27.2 years, three females) were scanned.

Dynamic GE BOLD images were acquired with a multiband EPI sequence while participants completed one localizer and one communication run in the scanner (2 × 2 × 2 mm^3^, 42 slices, FOV = 200 × 200 mm^2^, TE = 20 ms, TR_vol_ = 500 ms). In the localizer run, participants were explicitly instructed to perform mental drawing, mental calculation and inner speech interleaved with rest periods. In the communication run, participants encoded a six-letter word by performing the assigned mental task while the desired letter was highlighted. One communication run lasted eleven minutes.

**Fig. 3:**
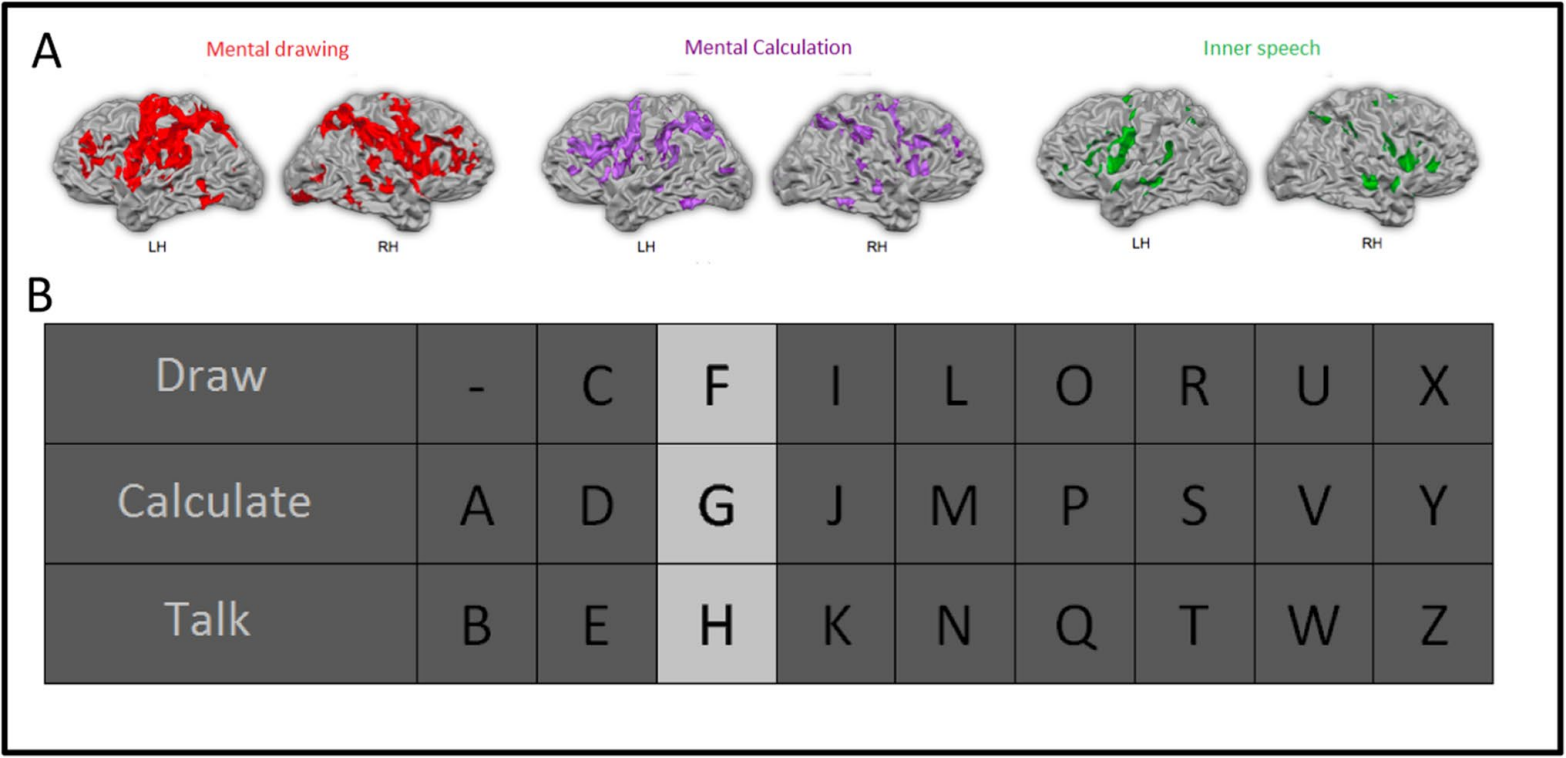
**A** Brain activation of an individual participant evoked by performing three different mental tasks. For every mental task, activations are shown for the left (LH) and the right hemisphere (RH). Every mental task evoked a unique brain-activation pattern that can be differentiated by fMRI. **B** The visual letter encoding scheme. Combining three mental tasks and nine different time intervals, the letter-encoding technique allows for encoding 3 × 9 = 27 different characters (26 letters and a blank space). Each column is highlighted for 10 s, during which the participant performs the corresponding mental task for the desired letter to be encoded

The decoding was performed using the real-time data analysis software package Turbo-BrainVoyager (version 3.2, Brain Innovation B.V., Maastricht, The Netherlands). For a systematic analysis, the software was used in offline mode. However, data analysis is possible (and was informally done in a few participants) also in a real-time fashion. The signal from the network of activated voxels for each mental task (Fig. 3) in the localizer run were then sent to the automatic BOLD decoder algorithm. The decoding accuracy was determined as the letter-position score per encoded word, which would add up to six if all letters were correctly decoded as first letter choice and 12 in case all encoded letters appeared as the 2nd letter choice. Across participants, all words were correctly decoded with a mean letter-position score of 10.4 (SD =  ± 4.8) constituting an excellent result. This study is currently in preparation for publication.

In conclusion, distinguishing three different mental tasks is feasible at 9.4 T with high decoding accuracy based on single-trial BOLD signals using moderate spatial resolution (2 mm isotropic) and whole brain acquisitions.

## Visual and auditory neuroimaging at 9.4 T

Kemper et al. investigated the improvement in CNR afforded by 9.4 T fMRI by comparing the variance explained by a Gaussian population receptive field model of responses in visual cortex [56]. Using an 8-channel transmit/24-channel receive surface coil (Life Services, Minneapolis, MN, USA), non-subject-specific B_1_^+^ phase shim [57] and GE EPI, T_2_*-weighted functional images were obtained at 1.05 mm isotropic (for more details see—[56]). Using the same stimuli, similar imaging settings (1.1 mm isotropic at 7 T) and matched processing and analysis, the median explained variance *R* values in 9.4 T participants was found to be significantly higher than at 7 T in V1 and V2 (*R* = 0.68 ± 0.07 at 9.4 T vs 0.52 ± 0.10 at 7 T).

T_2_*-weighted functional data suffer from vascular biases that render their interpretation at the level of layers (and columns) cumbersome. For this reason, other approaches (T_2_-weighted fMRI, CBF or CBV) have been proposed that generally trade SNR for specificity. Moving to magnetic fields higher than 7 T can benefit these applications, as demonstrated by early work at 9.4 T conducted in Maastricht using 3D GRASE [58, 59], in which Kemper and colleagues were able to unveil ocular dominance columns at an isotropic resolution of 0.7 mm isotropic [56].

**Fig. 4:**
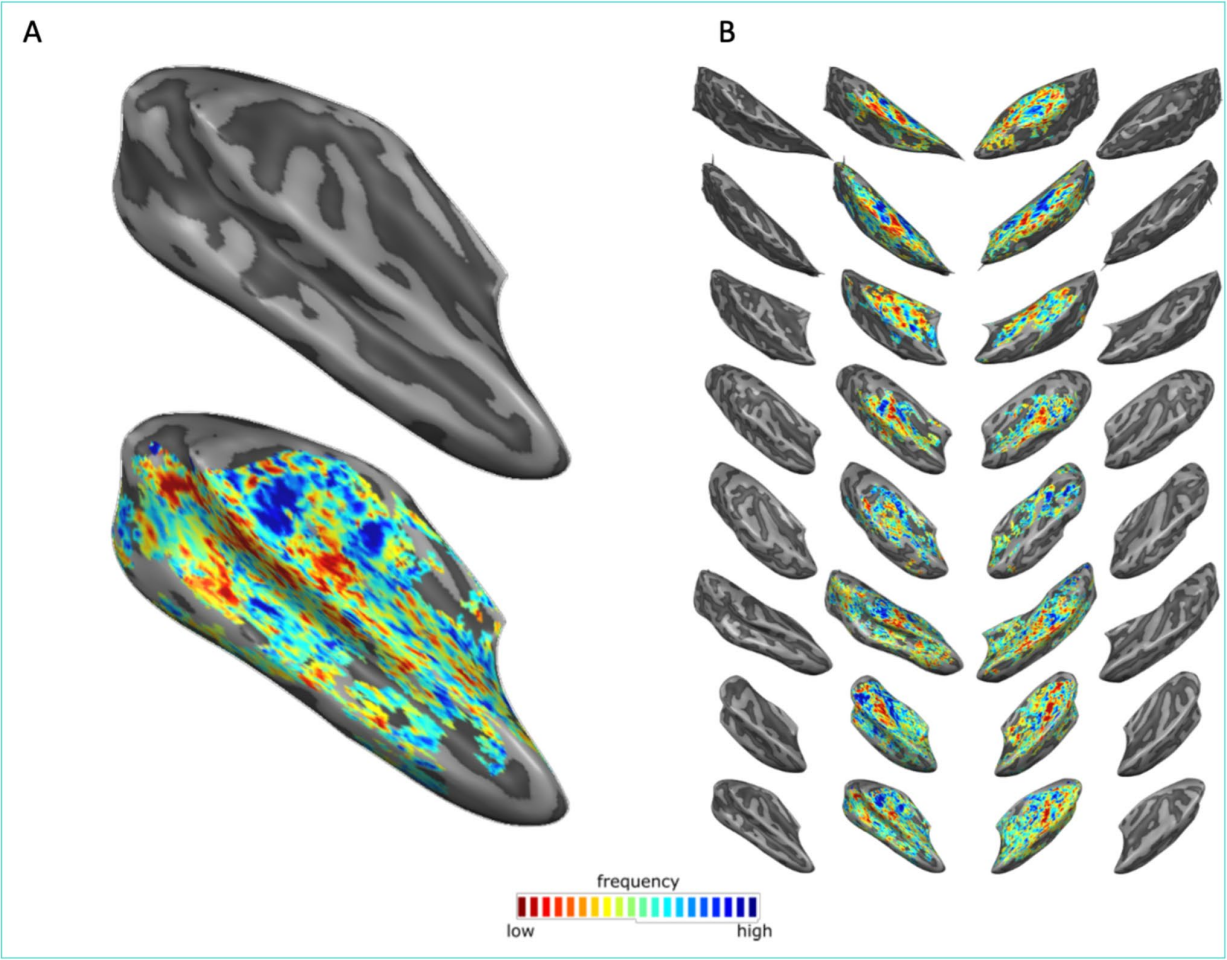
Tonotopic maps in temporal cortex obtained with GE EPI. **A** The right hemisphere surface of the temporal lobe (top) and tonotopy (bottom) of one of the volunteers. **B** Tonotopic maps (and cortical surfaces) of eight volunteers

Beyond applications targeting visual cortical regions, in Maastricht the 9.4 T scanner has been used to investigate the cortical organization of temporal (auditory) regions using a dedicated head RF coil (Life Services LLC, Minnesota, MN, USA) (8 channels Tx and 32 channel Rx). Preliminary results of data collected by driving the eight transmit channels with a constant, non-subject specific RF shim, and the CMRR multiband EPI sequence with FLEET reference line acquisition is presented here [60] (48 transversal slices, TE/TR/TA = 17/2400/1200 ms, silent gap 1200 ms for stimulus presentation, matrix size 156 × 200, phase-encoding direction anterior–posterior, 0.8 mm isotropic nominal resolution, Multi-band/GRAPPA-Factor 2/3, nominal flip angle 90°). To demonstrate the feasibility of acquiring functional (T_2_*-weighted) data at high isotropic resolution at 9.4 T, we measured auditory cortical responses elicited by presenting tones of different center frequency (tonotopy mapping: 9 center frequencies presented in blocks, center frequencies were log spaced between 200 Hz and 6 kHz). The resulting tonotopic maps (Fig. 4—best frequency coding) follow the established pattern for frequency preference in temporal regions (i.e. a high-low–high frequency gradient (see [61] for more information on how to interpret tonotopic maps)).

Taken together, these examples showcase how the higher BOLD CNR attainable at 9.4 T allows for routine experiments at relatively high (1.05 mm isotropic) resolution, which result in more statistical power for, e.g., computational modeling. At the same time, this additional SNR can be beneficial to high resolution investigation at submillimeter level that probe the mesoscopic architecture of the cortex.

## Layer-specific VASO at 9.4 T

Functional blood volume imaging with VASO [62, 63] can benefit twofold from imaging at 9.4 T: (A) the supralinear increase of SNR [2] allows higher resolutions in the thermal noise dominated regime of submillimeter voxels. (B) The longer T_1_ values and the correspondingly long-lasting inversion-recovery of the longitudinal magnetization provides more time for the blood to refill the vasculature with non-inverted spins, enhancing the contrast. However, in order to apply VASO on a human 9.4 T scanner, numerous technical limitations must be accounted for, for instance as follows: (1) SAR constraints, (2) B_1_^+^ inhomogeneity, (3) limited inversion efficiency, (4) strong BOLD contaminations, and (5) faster T_2_^*^ decay during the readout.

In 2016/2017, we conducted a study of CBV-sensitive fMRI methods at 9.4 T with the goal to assess the feasibility of sub-millimeter resolution depth-dependent fMRI in light of these challenges. We performed seven in vivo experiments with a dual-row 16-channel Tx/31-channel Rx array coil [32]. The aforementioned challenges were addressed as follows: (1) conservative SAR limits were adhered to by exploiting the lower flip angles of a 3D EPI readout and refraining from conventional 2D EPI readouts [47]. (2) B_1_^+^ was homogenized with static phase shimming, using the workflow and parameters described in Sect.  pTx workflow [37]. (3) Inversion efficiency was kept above 85% by customizing an adiabatic TR-FOCI pulse [46] for lower SAR at 9.4 T, including an increased adiabaticity with a reduced bandwidth and phase skip of 30º [64]. (4) The BOLD effect was assessed and removed from VASO with an interleaved acquisition approach every 1.5 s [64]. (5) Fast EPI readout was enabled by the high-performance head gradients. The sequence parameters were: TE = 21 ms, in-plane resolution 0.74 mm, nominal slice thickness perpendicular to the cortex 1.5 mm, TI_1_/TI_2_/TR = 1200/2700/3000 ms assuming blood T_1_ = 2300 ms [65]. Activity was assessed during a 12-min finger tapping task (30 s act vs. 30 s rest). With this setup, most of the challenges specific to 9.4 T VASO could be successfully addressed.

**Fig. 5:**
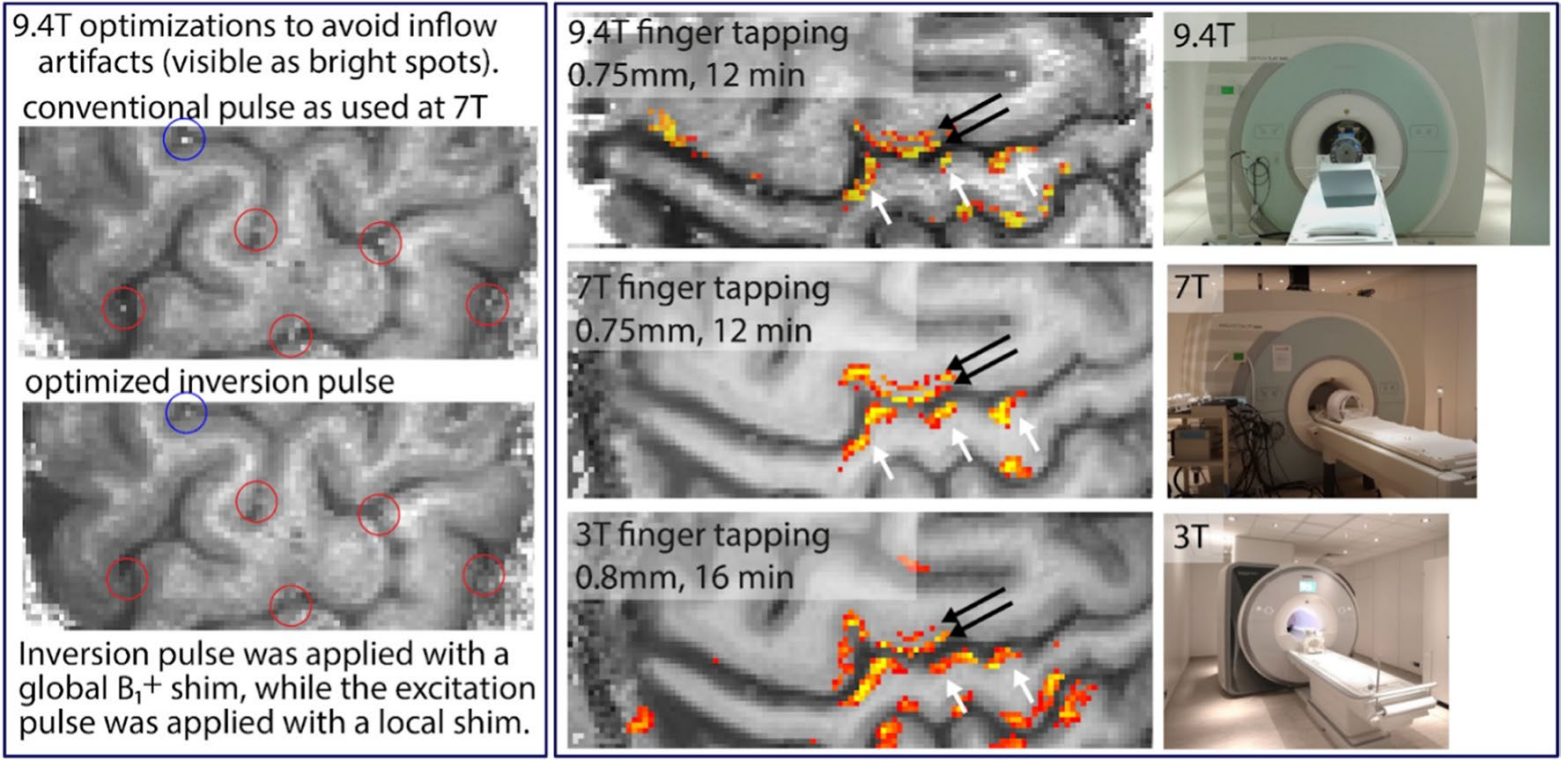
The left panel highlights one of the most important challenges of VASO at 9.4 T., namely obtaining a VASO T_1_ contrast without inflow of fresh (un-inverted blood). We achieve this with an alternating B_1_^+^ shim (for inversion and excitation pulses, respectively), and by means of pulse optimizations including phase skips and SAR-optimized lower bandwidth. The right panel depicts representative high-resolution maps of CBV changes. 9.4 T can reveal the same laminar activation features as lower field strengths

High-resolution maps of CBV changes and BOLD signal show promising detectability of cortical depth-dependent response variations (Fig. 5). The results demonstrate that VASO at 9.4 T is feasible and can be a useful tool for activity mapping in the mesoscopic regime of the human brain. For a more in-depth discussion of this work, see [66].

## Columnar human fMRI

**Fig. 6:**
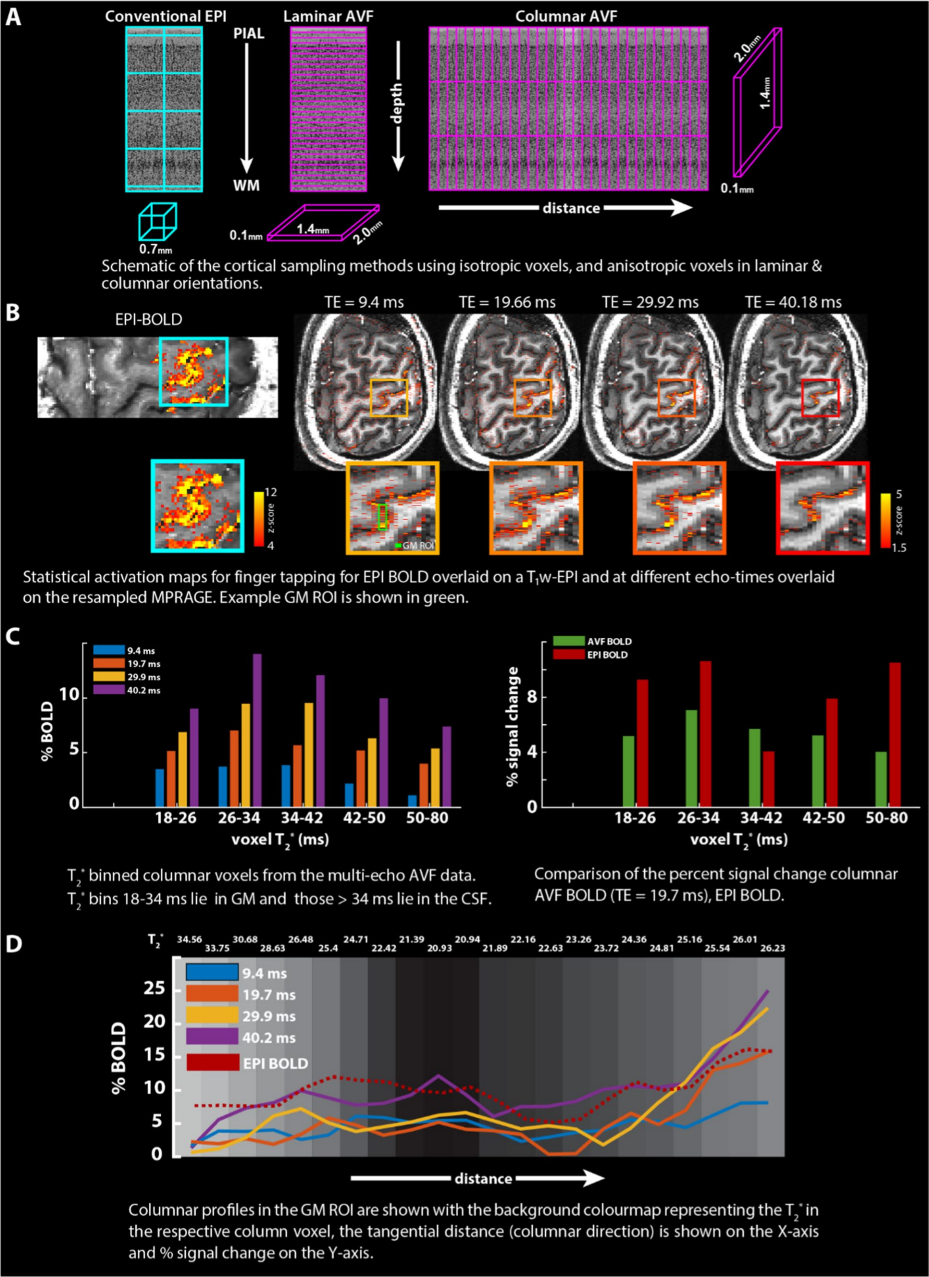
**A** Schematic representation of the conventional isotropic and the anisotropic sampling schemes of cortex illustrating the differences for laminar and columnar acquisitions, **B** example Z-score maps and zoomed panels for the EPI-BOLD and anisotropic multi-echo BOLD data for the finger-tapping experiment overlaid on a registered T_1_-weighted image at each TE. Bar plot of % signal change with voxels binned according to different T_2_* for the four TEs of the AVF-BOLD data, (**C**, left) and comparable TE = 19.7 ms, EPI-BOLD (**C**, right). **D** Columnar profiles of the % BOLD signal change at different TEs from the AVF data and the EPI-BOLD data in the GM ROI (zoomed cyan panel of **B**) overlaid on colourmap corresponding to T_2_* of the voxel.

Cortical layers occupy a much finer spatial scale than voxel sizes that are typically used at UHF and such voxels are inadequate to directly sample the depth-dependent fMRI signal. While isotropic voxels have their advantages, anisotropic voxels can be used to sample the dimension-of-interest, laminar or columnar, more finely. Although CBV-weighted fMRI using VASO at UHF [66, 67] provides more specificity for applications targeting laminar and columnar investigations of the cortex, work from Kashyap et al. have shown that BOLD fMRI with ultra-high spatial resolution of 0.1 mm along the laminar and columnar direction (Fig. 6A) [68] can also provide increased spatial localisation. In this study, the feasibility of columnar human fMRI is demonstrated using anisotropic voxel FLASH (AVF) for multi-echo BOLD fMRI at 0.1 mm resolution [69].

The B_0_ and B_1_^+^ fields were homogenized using the procedure outlined in Sect.  pTx workflow above. After satisfactory shims were obtained, a T_1_-weighted anatomical MPRAGE (0.6 mm isotropic, TE/TI/TR = 3.64/1200/3750 ms, *α* = 5°, GRAPPA = 3, Partial Fourier slice = 7/8, TA = 9 min) was acquired followed by one functional run of anisotropic voxel FLASH (AVF) (0.1 × 1.4 × 2.0 mm^3^, TE_1_/TE_2_/TE_3_/TE_4_/TR = 9.4/19.6/29.9/40.1/2100 ms, GRAPPA = 2, TA = 14 min) (Fig. 6B). In this study, one functional run of SS-SI-VASO (0.74 × 0.74 × 1.70 mm^3^, TI_1_/TI_2_/TE/TR = 50/1025/21/1623 ms, GRAPPA = 2, Partial Fourier phase = 6/8, TA = 13 min) was also acquired. For the full list of sequence parameters, please see [68] and [66]. Acquisition slab was positioned perpendicular to the surface of M1 and the single-slice anisotropic acquisition was positioned using the MPRAGE as the anatomical reference.

Data were pre-processed using SPM12, ANTs and ITK-SNAP. Additionally, the multi-TE data were used to fit a mono-exponential decay to compute a T_2_* map. Statistical analyses were carried out in FSL FEAT (for details on data processing see [68] and [66]). ROIs were manually defined on the hand knob (Fig. 6B, cyan zoomed panel) and columnar profiles were extracted using MATLAB.

Robust statistical activation was obtained using both the VASO and the multi-echo BOLD AVF datasets. The VASO-CBV results were comparable to those described in Sect.  Layer-specific VASO at 9.4T and, therefore, the VASO-BOLD (referred as EPI-BOLD) and the multi-echo AVF BOLD data will be discussed here (Fig. 6B). The statistical values in the AVF data were smaller than the EPI-BOLD, due to higher SNR in the EPI-BOLD dataset owing to its increased voxel volume (3.3 times that of the AVF data). The multi-echo dataset allowed us to compare the %BOLD signal change from individual TEs and bin them with respect to the voxel’s T_2_* in M1 (Fig. 6C, left). We find that the %BOLD signal increases with increasing TEs in all voxels and largest activation was found with the longest TE in T_2_* bins (18–34 ms). Given that the intravascular signal at 9.4 T is virtually nonexistent [4] even for short TEs, this large activation can be attributed to increased contribution from the extravascular static dephasing effects from the CSF compartment due to partial voluming [70, 71]. The partial volume effect is further emphasized when the same analysis was done (Fig. 6C right) comparing AVF-BOLD at TE≈19.7 ms (green) with the EPI-BOLD data (red) where the largest %BOLD signal for the EPI-BOLD data was found in the 26–34 ms as well as in the 50–80 ms T_2_* bins. Figure 6D shows plots of columnar BOLD signal profiles in the hand knob region (Fig. 6B green ROI) with the background colourmap indicating the voxel’s T_2_* (in ms). We capture mesoscale TE-dependent BOLD signal changes in GM voxels along cortical distance (orthogonal to cortical depth i.e., columnar direction). The large %BOLD changes over relatively short cortical distance (0.2–0.4 mm typically unresolved using conventional EPI-BOLD) are consistent with results in the visual cortex (Fig. 6 in [67]). We also observed the absence of a clear relationship between the voxel T_2_* and its measured BOLD signal change indicating a broad sensitivity for the BOLD contrast. These plots also suggest the feasibility of adopting TE > GM T_2_* is an important finding for high-spatial resolution imaging using EPI for columnar or laminar imaging at UHF.

Taken together, although VASO-CBV weighted data can provide superior spatial specificity to activation compared to the BOLD signal weighted data [67], spatial specificity of the BOLD contrast can be improved by increasing its spatial resolution such as using AVF-like acquisitions [68]. AVF-like approaches and extensions with multi-echo readouts [72, 73] and modelling approaches [74, 75] provide us with avenues for further understanding of the physical and physiological bases of the BOLD signal as it remains the workhorse contrast for fMRI.

## Post-mortem human brain imaging at 9.4 T

**Fig. 7:**
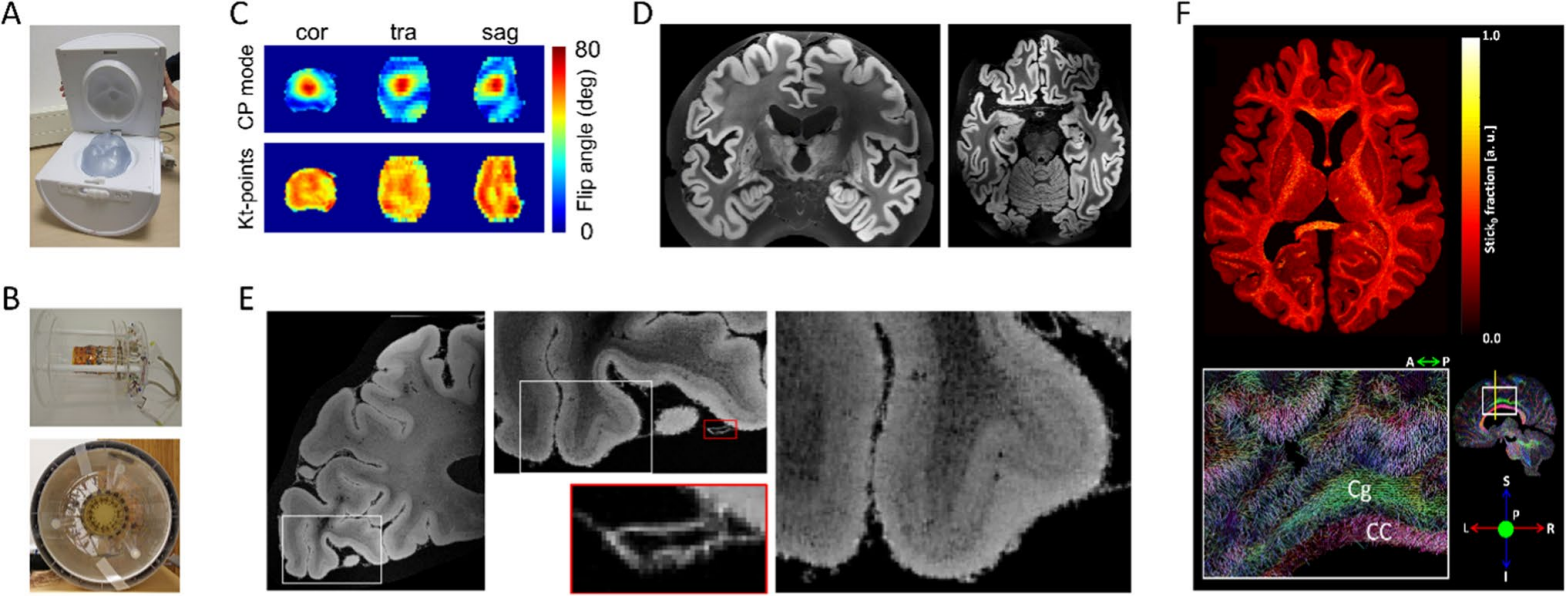
**A** The custom-built 8Tx/24Rx 9.4 T whole post-mortem human brain RF-coil. **B** The custom-built 16Rx 9.4 T medium-sized post-mortem sample RF-coil outside of (top) and inside (bottom) the 16Tx ring **C**) B_1_^+^ maps showing transmit efficiency and homogeneity over the whole post-mortem brain with substantial inhomogeneity in standard circularly polarized (CP) mode, which is improved greatly with k_T_-points transmit phases optimized for homogeneity. **D** A coronal (left) and transverse slice (right) through early whole post-mortem brain 3D GE acquisitions at 200 µm isotropic. **E** Transverse slices through a GE acquisition at 60 μm isotropic of an occipital lobe sample in the medium-sample coil at different zoom levels. **F** k_T_-dSTEAM 400 μm diffusion results: a transverse slice through the Stick0 fraction map resulting from a Ball&Stick model fit (top) and direction color coded DTI primary eigenvector in a sagittal slice with zoom-in (bottom) *CC* corpus callosum, *Cg* cingulum

Small post-mortem human brain tissue samples can be used on pre-clinical MRI systems (animal scanners or spectroscopy systems) to examine and investigate fundamental neuroanatomy questions at the mesoscale [76–79]. However, small bore scanners are limited to small tissue samples, for example on the order of millimeters. From about 2015 onward, the computational brain connectivity lab at Maastricht University undertook a program of RF coil construction and MR method development to investigate the limits of contrast and resolution on the 9.4 T large bore system for much larger human brain samples. The focus was on gradient-echo and diffusion imaging of post-mortem whole human brains and intact post-mortem human occipital lobes. Previous work had already shown that resolutions considerably superior to that achievable in vivo are attainable at 3 T and 7 T [80–82]. Limiting factors in optimizing acquisitions further and using the advantages of 9.4 T were as follows: limited large-bore gradient performance, non-optimized RF coils for the intended samples, and RF field inhomogeneity over the brain at 9.4 T considerably higher than at 3 T or 7 T. For the RF coils, we set out to design and build several special purpose postmortem tissue coils, prominent among which are a 9.4 T 8-channel parallel transmit (pTx), 24-channel receive RF coil for whole post-mortem human brains (Fig. 7A) and a 16-channel cylindrical RF coil to image medium post-mortem occipital lobe samples (~ 80 × 80 × 80 mm^3^, Fig. 7B, [83]).

Mitigating the severe transmit (B_1_^+^) field inhomogeneity over the entire brain samples at 9.4 T could be dealt by using kt-points composite excitation pulses (Fig. 7C; [37, 44] and modifying kt-point refocusing pulses for use in diffusion weighted STEAM imaging [84]. Using these techniques, early 3D GE acquisition results quickly provided anatomical whole brain images at 200 µm isotropic and better (Fig. 7D; [85, 86]). For occipital lobe imaging 3D GE acquisition results could be brought beyond 100 µm isotropic up to 60 µm isotropic (Fig. 7E) with quantitative T_2_* and T_2_*-weighted contrast providing cortical layer specific details, such as the stripe of Gennari ([83] for further results and details).

For diffusion MRI (dMRI) in particular, restricted large-bore gradient performance is a limiting factor in combination with the decreasing T_2_ with increasing B_0_ as well as further decreases in water’s apparent diffusion coefficient (ADC) and T_2_ in fixed tissue [87]. The applied solutions were modifying k_T_-point pulses for use in diffusion-weighted STEAM imaging, creating k_T_-dSTEAM [84], achieving B_1_^+^ homogenization across whole human brain specimens and smaller sections and using the increased T_1_ with increasing B_0_ to compensate for decreasing T_2_ in diffusion weighting. Using kT-dSTEAM, ultra-high isotropic resolution data (400 μm, Figure F) at moderate *b*-values (3000 s/mm^2^) [84] and a high resolution (1000 μm) whole brain data at high *b*-value (6000–8000 s/mm^2^) could be obtained [84].

## Practicalities, reflections and lessons learned

At our site, we have the fortune of having human 3 T, 7 T and 9.4 T scanners available under one roof. The high-end 3 T is a robust machine for most neuroscience and clinical applications, and the 7 T is for the more demanding neuroimaging and research applications at UHF. The 7 T has been well-aligned with the rest of the Siemens UHF community, which allowed for active sharing of protocols, sequences, and tools. It has served us well and can be considered as an essentially push-button workhorse that can be run by neuroscientists and clinical researchers with minimal or no support from radiographers or physicists. This experience has been quite different for the 9.4 T, for which several reasons can be identified.

A key choice was made regarding the 9.4 T operation when it was commissioned in 2013: it was decided to always operate in parallel-transmit mode (initially with 8, later 16 transmit channels), instead of having the custom coils prepared for single-channel use, e.g. with power splitters. In hindsight, this was a poor choice. The reasoning at the time was to get the highest possible additional benefit from 9.4 T over our other scanners: highest-resolution (functional) imaging, short echo-planar readouts thanks to the more powerful head gradients, combined with dedicated RF coils, and B_1_^+^ shimming or full pTx. The disadvantage of running in pTx mode configuration, also when actually not exploiting pTx capability, is the considerably less operator-friendly setup, system instability in terms of frequent occurrence of software errors which meant that the system remained largely inaccessible to non-expert users. For many use cases, the additional required effort, patience and resilience at 9.4 T thus resulted in a relatively incremental or diminished return on investment, compared to choosing a session on the push-button 7 T with guaranteed success within the scheduled scan time. A better strategy would thus have been to invest first into enabling single-channel operation of the 9.4 T, in order to make it identical to the 7 T in terms of software and operability, and thereby allowing easy transition of researchers and radiographers between the systems without expert support. Building on broad user acceptance, the curiosity and interest to explore its more demanding capabilities would have potentially resulted naturally. Furthermore, despite good collaborative ties to other sites, we found that being at 9.4 T with pTx made it a lot harder to benefit from solutions in the community and required considerable in-house effort, some of which has been described above.

The choice of a head gradient system for the (otherwise whole-body) 9.4 T scanner further resulted in a rather unique system. With its slew rates exceeding those at our 7 T it has enabled echo-planar and diffusion-weighted imaging that might otherwise have been impracticable in the battle for shorter echo times, but it also posed additional practical, albeit not per se field strength related challenges. This included the tight space constraints for RF coils, connectors and boxes that have to be fit without obscuring the already limited visual angle, and the observation of the well-known shoulder in-folding artifacts when imaging the whole brain. More than initially expected, we were also limited by gradient heating for high-resolution EPI and high *b*-value DWI. The burning of one gradient set prompted us to find solutions including the placement of additional (optical) probes for temperature monitoring, and suitable schemes for cycling diffusion directions to share the heat load across the different gradient axes. Despite the attractive capabilities of the head gradients, a future system upgrade will likely include a swap to body gradients, not least to open up the system to non-neuro applications such as musculoskeletal MRI that have meanwhile gained importance in Maastricht.

Due to the situation described above came the need to build up a breadth of expertise in a small team of physicists and support staff, with only a few people from different groups and with distinct goals, we worked on sequence programming, implementation of the B_0_B_1_ toolbox, RF pulse design, coil conceptualization and simulation, coil building and validation. As such, we were rendered vulnerable to the departure of team members and critical loss of momentum on several accounts. The 9.4 T scanner service provided by the vendor did not differ from that of the 7 T scanner, and there was no dedicated site scientist employed by the vendor. Nevertheless, Maastricht has benefited enormously from the presence of a highly experienced staff scientist (co-author CJW) for system support, debugging and upkeep, which arguably saved us from making many service calls to the vendor that would otherwise have been necessary.

Considering the latest software and hardware pTx capabilities of 7 T scanners, these will certainly lead to substantial improvement in the user-friendliness and potential of the 9.4 T scanner including its pTx capabilities. A similar upgrade as for our 7 T is in planning, and this is expected to address many of the practical software-related limitations described above. Importantly, it will also (re)align the software between the 9.4 T and the 7 T.

With regards to subject inclusion, the same criteria as at the 7 T are applied (with few exceptions like dental implants and retainers). There are no restrictions on how often a subject can be scanned. Also, the duration of the in vivo scan sessions is not explicitly restricted, but it does not typically exceed two hours, depending on the experience of the subject. Even though the subject experience is usually not recorded with a questionnaire, subjects (including MR-naïve ones) have reported only mild and short-term scanner-related side effects such as vertigo, metallic taste, nausea, and headache. First epilepsy patients have been scanned at the 9.4 T as part of a recent study, but the overwhelming majority of subjects until now have been healthy volunteers.

## Discussion

The overview presented here highlights a broad range of applications that could benefit from fields above 7 T. For some of the presented projects further SNR and resolution gains at 9.4 T remain possible. Most of the applications will likely experience compounded benefits at even higher fields but both these undertakings will require significant additional development, for instance to overcome the coverage restriction of VASO due to SAR. One obstacle that has been largely overcome is the B_0_-sensitivity of EPI readouts, which remain the workhorse for functional and diffusion applications, thanks to advancements in GRAPPA auto-calibration scans and incorporation of pTx support. The overall gain in SNR observed is impressive but hinges on optimized coils and specialized techniques (acquisition and contrast) to be utilized completely. With increasing field strength, dedicated acquisition approaches and/or region-specific coils will become even more important.

The largest remaining potential for 9.4 T imaging is in pushing the limits of spatial resolution. On the hardware side, even more powerful gradients that are wider in diameter will enable a broader set of RF coil configurations as well as the possibility of highly parallelized receive arrays. Further methodological developments will be focused on gaining additional SNR for routine (whole brain) functional and structural imaging. This can be achieved with continued optimization and extension of the presented workflows or the adaptation of approaches such as universal pulses [88] that are now becoming rapidly established on the new 7 T platforms. The ex vivo imaging pipeline has enabled a continuing flow of applied investigations involving pathological conditions (e.g. Parkinson’s Disease) and different species (e.g. seal). More in-depth investigations of specific human brain systems, for instance the early visual system, are also ongoing. Furthermore, the addition of further specialized ex vivo coils, such as one specific to thick brain tissue slabs, is also envisaged to open further possibilities.

We believe that our experience in optimizing various acquisition approaches for 9.4 T and combining them with parallel transmission have paved the way towards further neuroscience applications, including the first exploration of its clinical use in epilepsy patients. As one of the few > 7 T systems worldwide, it also plays a bridging role towards even higher field strengths, for example in the AROMA project (11.7 T at CEA Neurospin). Similarly, as the currently highest field strength system in the Netherlands it will act as test platform in preparation for the recently funded 14 T scanner that will be installed at Radboud University Nijmegen and become available to the Dutch high field community in the coming years.

## Data Availability

The authors do not have a signed data sharing agreement from the participants of this study. Therefore, as per the guidelines of the Ethics Review Committee Psychology and Neuroscience (ERCPN) at Maastricht University, we are unable to make their data publicly available. The ethics committee can be reached at ercpnfpn@maastrichtuniversity.nl.
